# Climate change and professional responsibility: a cross-sectional survey among German general practitioners

**DOI:** 10.1186/s12910-026-01516-1

**Published:** 2026-06-09

**Authors:** Niklas Freese, Anja Hesse, Bjarn-Ove Tetzlaff, Sabine Salloch

**Affiliations:** 1 Hannover Medical School, Institute for Ethics, History and Philosophy of Medicine, Carl-Neuberg-Str.1, 30625 Hannover, Germany; 2 Hannover Medical School, Institute of General Medicine and Palliative Medicine, Carl-Neuberg-Straße 1, 30625 Hannover, Germany; 3 Department of Psychosomatic Medicine and Psychotherapy, Hannover Medical School, Carl-Neuberg-Straße 1, 30625 Hannover, Germany

**Keywords:** Climate change, General practice, Primary care, Medical ethics, Planetary health, Environmental sustainability, Clinical decision-making, General practitioners, Cross-sectional survey, Germany

## Abstract

**Background:**

Climate change increasingly affects population health and healthcare systems. While normative frameworks emphasize physicians’ societal role in climate protection, empirical evidence on how general practitioners perceive and address climate- and environment-related issues in daily clinical practice remains somewhat limited and not yet fully consistent across studies.

**Methods:**

We conducted a nationwide cross-sectional online survey among general practitioners (*N* = 500; 38.2% female) in Germany. The questionnaire assessed attitudes toward climate protection, attribution of responsibility for climate- and environment-related practice in healthcare, and the frequency of addressing climate- and environment-related issues in practice by patients and practitioners. Data were analyzed using descriptive statistics, group comparisons, and correlation analyses.

**Results:**

A majority of respondents (65.8%) considered climate- and environment-related issues relevant to their professional role and endorsed physicians’ function as societal role models. Agreement regarding individual responsibility within clinical practice was comparatively moderate (22.6%), with responsibility more frequently attributed to institutional (29.1%) and policy levels (49.2%). Attitudes toward environmental responsibility were largely consistent across subgroups defined by federal state, community size, practice type, and professional experience, while female general practitioners reported higher levels of environmental concern (M = 3.94 vs. M = 3.43; *p* < .001), indicating a small to moderate effect. Climate- and environment-related issues were reported to arise regularly in general practice, initiated by both physicians and patients (monthly or more often in physicians 68.4% versus patients 59.2%; never in physicians 12.4% versus patients 12.6%).

**Discussion and Conclusions:**

The findings suggest that German general practitioners broadly acknowledge the relevance of climate- and environment-related issues to their professional role and are open to integrating such considerations into clinical context. At the same time, ambivalence regarding individual responsibility highlights the need for clearer normative orientation. Integrating climate- and environment-related considerations into professional standards, clinical guidelines, and medical education may help provide such orientation and support physicians in navigating ethical tensions.

**Supplementary Information:**

The online version contains supplementary material available at 10.1186/s12910-026-01516-1.

## Background

Climate change is increasingly recognized as a fundamental threat to global health. It leads to rising average temperatures, more frequent extreme weather events, and profound changes to ecological systems that affect human health in complex ways [1, 2]. For instance, an increased number of heat-related deaths has been identified to be directly attributable to anthropogenic climate change [3]. In addition, the main health consequences include exacerbation of cardiovascular and respiratory diseases and accelerated spread of vector-borne infections [4]. Moreover, climate change gives rise to indirect consequences, such as food insecurity and reduced mental health [5]. Vulnerable population groups, including older people, children, and those belonging to socioeconomically disadvantaged communities, are particularly at risk, as studies indicate that this exacerbates existing health inequalities [4]. The World Health Organization emphasizes the systematic integration of health into climate protection strategies, describing this as a health policy imperative [6].

The healthcare sector itself is a significant driver of climate change. It accounts for around 5% of global greenhouse gas emissions [7]. Emissions arise from both direct sources within healthcare facilities and indirect sources across the broader supply chain [8]. In hospitals, operating theatres generate 30–70% of total hospital emissions, primarily due to disposable materials and energy-intensive processes [9]. In general practice, the composition of emissions differs from that of hospital care [4, 10, 11]. Evidence suggests that patient and staff transportation, along with heating, constitute the primary sources of the carbon footprint in the outpatient sector [10]. However, the contribution of pharmaceuticals remains difficult to quantify [12].

Efforts to mitigate the healthcare sector’s environmental impact have emerged at multiple levels. At the system level, national health systems have committed to *net zero* strategies [13]. Within healthcare institutions, reduction strategies include waste separation and recycling, as well as telemedicine [9, 14]. Initiatives promoting “Green Hospital” designs advance sustainable practices to foster a more resilient healthcare [15]. At the individual level, physicians and non-physician healthcare professionals can contribute through sustainable prescribing, the avoidance of unnecessary diagnostics, and support for patient choices favoring low-carbon treatments. However, a profound transformation is still required, including reducing oversupply, relocating inpatient services, ensuring sustainable procurement and decarbonizing supply chains [7].

In general, it is stressed that systemic approaches including multi-level governance and allowance for socio-ecological interaction are needed for appropriately dealing with adaptation and mitigation in climate change [16]. In climate ethics, however, also issues of individual responsibility are problematized, e.g. in highlighting the missing effect of individual climate action [17, 18]. The ethical discourse on how responsibility for climate protection in health care should be understood and distributed remains unresolved [19]. The concept of responsibility as such and its applicability to climate protection is subject to ongoing philosophical debates even with recent proposals also to allow for a “de-responsibilisation” under certain circumstances [20]. At the same time, other approaches emphasize that responsibility in the context of climate change is not limited to individual actors, but is shared across different levels, including social and institutional structures [21]. Regarding health care, one main line of argument locates primary responsibility at the systemic and institutional levels. It is argued that achieving effective decarbonization requires structural reforms and political action rather than relying on individual behavioral change [22, 23]. By contrast, the *Planetary Health Pledge*, for example, follows the Declaration of Geneva in formulating individual physician’s responsibility towards environmental protection and social justice [24, 25]. Climate protection has also been linked to the professional identity of medical practice, with proposals to integrate ecological dimensions into ethics consultation and curricula [26, 27]. These considerations become particularly relevant in clinical practice, where ethical reflection is translated into concrete decision-making between physicians and patients. In this context, calls have been made to incorporate ecological considerations into patient information, e.g. through *Green Informed Consent* [28]. Conversely, it is argued that there is no general duty to inform, in order to avoid manipulation and preserve trust [23]. Patients’ ecological preferences may be considered and respected as part of their autonomy [29]. A systematic review indicates that this debate is still in its early stages and that clearer guidance on the distribution of responsibility is needed [30].

There is a growing body of empirical research examining how healthcare professionals, including GPs, perceive the relationship between climate change and health and their role in addressing it in clinical practice. Healthcare professionals widely recognize climate change as a relevant health concern; however, they often perceive their options for action to be constrained by time limitations, inadequate resources, and insufficient institutional support [31, 32]. Surveys among GPs show that many are willing to address climate-related health issues in clinical practice, while at the same time reporting substantial barriers such as lack of time and insufficient guidance [33] Similar observations have been made in German healthcare settings, where climate-related topics are only inconsistently addressed in routine care [34]. Institutional and organizational factors further shape these limitations. Surveys in German hospitals have identified sources of emissions, while emphasizing that the primary responsibility remains with the provision of optimal care [35]. Concurrently, evidence shows that sustainability committees can significantly enhance knowledge and commitment within hospitals [32]. Furthermore, surveys of medical professionals highlight the existence of ethical tensions. The propensity to embrace more environmentally sustainable, though potentially less effective, treatment modalities tends to increase as the tangible impacts of climate change on patients become evident [36]. These tensions are reflected in clinical interactions. Vignette studies demonstrate that sustainable therapy recommendations have a largely unchanged impact on patient satisfaction in cases of mild illnesses yet are subject to a significantly more negative evaluation in cases of severe illnesses [37]. Moreover, consultations that integrate both climate- and health-related arguments are less accepted than those focusing exclusively on health, a phenomenon that is more common among individuals who are skeptical of climate change [38]. However, existing studies often rely on broad and partly overlapping constructs such as awareness, perception, or motivation, which are not consistently defined and limit comparability across studies. This lack of conceptual clarity makes it difficult to systematically assess how healthcare professionals understand their professional role in the context of climate change.

Against this background, there is still limited empirical evidence on how specific stakeholder groups, particularly general practitioners, conceptualize their professional role and responsibility in the context of climate change. In addition, the outpatient care setting has received less attention compared to hospital-based research. We therefore conducted a cross-sectional survey study including a fictional case vignette with general practitioners (GPs) in Germany. GPs represent the third largest group of practicing physicians in Germany—after internists and physicians in training—and provide care for the entire population, not only for disease-specific patient groups [39]. Their position within primary care allows them to directly observe health effects of climate change and to operate at the intersection between individual treatment and population-level prevention. This specific characteristic of GPs’ practice is particularly relevant because medical responsibility has often been attributed to the well-being of individual patients, whereas climate protection requires a collective and long-term perspective. It remains rather unclear to what extent GPs are willing to incorporate ecological considerations into clinical decision-making or whether they rather regard this as a task for political and institutional actors. Our survey therefore aims to explore GPs’ attitudes and clinical experience with the main focus on their perception of individual responsibility in climate action. More specifically, it focuses on how GPs perceive the relevance of climate change for their professional role, how they attribute responsibility across different levels, and how climate- and health-related issues are addressed in everyday clinical practice, as well as how this relates to clinical decision-making. In doing so, our survey contributes empirical data to the ongoing ethical debate on sustainability and climate protection in healthcare and provides a basis for further ethical, educational, and policy-oriented reflection.

## Methods

### Procedure

The present study was designed as an exploratory, cross-sectional survey. Prior to data collection, an a priori power analysis was conducted using G*Power [40] to determine the required sample size for detecting medium effect sizes (Cohen’s f = 0.25) in a one-way analysis of variance (ANOVA) at a significance level of α = 0.05 and a statistical power of 0.95. The analysis indicated that a minimum of approximately 305 participants would be required to detect such effects. Before launching the survey, we performed a pre-test of the instrument. The questionnaire was reviewed by five GPs as members of the targeted group to ensure comprehensibility and clarity of the items and response options. They provided written feedback regarding minor clarifications and suggested adjustments, and the questionnaire was revised accordingly based on this feedback.

Survey participants were recruited via the DocCheck^®^ online panel on a best-effort basis, aiming for a final sample of approximately 500 respondents. Recruitment invitations were distributed electronically, and interested physicians in general medicine accessed the survey through a secure online platform. All participants received the same questionnaire in identical form. Participation was voluntary, anonymous and, compensated with €10. The study was approved by the Ethics Committee of Hannover Medical School (vote no. 11937_BO_K_2025).

### Materials

Data were collected using a self-developed questionnaire that was created in a multiprofessional process involving GPs, ethicists, and psychologists, with the aim of ensuring both clinical relevance and conceptual breadth. The questionnaire comprised four thematic sections (see *Supplement 1*). The first section presented a case vignette describing a fictional clinical scenario in which respondents evaluated statements concerning the balance between individual patient care and climate-conscious medical practice (see *Supplement 1)*. Specifically, the vignette involved the prescription of asthma inhalers as an example of a common therapeutic decision where both clinical and ecological considerations may play a role. The vignette was selected as a typical and clinically realistic decision-making situation in general practice, intended to reduce socially desirable responses and to capture ethical considerations as they may arise in everyday care. Responses were recorded on a 5-point Likert scale ranging from 1 (“strongly disagree”) to 5 (“strongly agree”). The second section focused on the frequency and manner in which climate-related topics were addressed in patient care, using a combination of single-choice items, frequency ratings, and open-ended questions. The third section assessed attitudes toward professional responsibility in the context of health versus environmental concerns, again using agreement ratings on a 5-point Likert scale. The fourth section examined participants’ endorsement or rejection of specific provisions from professional codes of ethics, such as the German Medical Association’s professional code of conduct and the Planetary Health Pledge, also using 5-point Likert items. Selected items across sections included optional free-text responses. Finally, the questionnaire collected demographic and professional background variables, including age, gender, years in practice, practice setting, community size, and German federal state. The selection of items followed an exploratory approach and was intended to capture different aspects of how GPs perceive and address climate-related issues in clinical practice, particularly in relation to the attribution of individual professional responsibility. This allows linking reported practices and attitudes to how physicians understand their professional role in this context.

### Statistical analyses

The data analyses were conducted using IBM SPSS Statistics (version 29.0.1; IBM Corp., Armonk, NY). Descriptive statistics and frequency distributions were calculated to characterize physician- and patient-initiated discussions on climate change and health. For selected survey items (ecological appropriateness of therapy decisions, familiarity with guidelines, and physicians’ role-model function) originally assessed on a 5-point Likert scale (1 = strongly disagree to 5 = strongly agree), responses were additionally recoded into three categories to facilitate interpretation of agreement patterns: values of 1 and 2 were grouped as disagreement, a value of 3 was treated as neutral, and values of 4 and 5 were combined as agreement.

To test for group differences, one-way analyses of variance (ANOVA) without repeated measures were conducted across federal states, practice settings, and community sizes; all assumptions for ANOVA were met. Gender differences were examined using independent-samples t-tests. Associations between continuous variables (e.g., age, years of professional experience, community size) and agreement were assessed with bivariate correlations. Effect sizes were determined using r-coefficient. Interpretation of effect sizes followed the benchmarks proposed by Cohen [41], with values of *r* = .10, 0.30, and 0.50 indicating small, medium, and large effects, respectively. Sample sizes varied slightly between analyses because cases with missing values were excluded listwise from the respective tests.

## Results

### Participants

A total of 500 participants took part in the study. All participants were board-certified GPs. The mean age was 54.20 years (SD = 11.04, range = 28–81), and the mean professional experience was 23.04 years (SD = 10.78, range = 1–50). Regarding practice setting, 210 (42.0%) worked in solo practices, 208 (41.6%) in joint practices, 30 (6.0%) in practice collectives, and 52 (10.4%) in medical care centers. In terms of gender, 191 (38.2%) identified as female, 305 (61.0%) as male, and 4 (0.8%) did not provide this information. Participants were distributed across all federal states of Germany, with the largest proportions practicing in Bavaria (*n* = 115, 23.0%), North Rhine-Westphalia (*n* = 89, 17.8%), and Baden-Württemberg (*n* = 64, 12.8%). Practice location size varied, with 74 (14.8%) working in places with fewer than 5,000 inhabitants, 144 (28.8%) in places with 5,000–19,999 inhabitants, 116 (23.2%) in places with 20,000–99,999 inhabitants, 73 (14.6%) in cities with 100,000–499,999 inhabitants, and 93 (18.6%) in cities with 500,000 or more inhabitants. Frequencies of physicians across practice settings, federal states, and town sizes are summarized in Table 1.

**Table 1:** Sample Characteristics

	**years**	**SD**
Age	54.20	11.04
Professional experience	23.04	10.78

	*n*	%
Female Male Diverse / No answer	305 191 4	38.2 61.0 0.8
Solo practice	210	42.0
Joint practice	208	41.6
Practice collective	30	6.0
Medical care center	52	10.4
Baden-Württemberg	64	12.8
Bavaria	115	23.0
Berlin	20	4.0
Brandenburg	9	1.8
Bremen	3	0.6
Hamburg	15	3.0
Hesse	52	10.4
Mecklenburg-Vorpommern	9	1.8
Lower Saxony	33	6.6
North Rhine-Westphalia	89	17.8
Rhineland-Palatinate	28	5.6
Saarland	7	1.4
Saxony	17	3.4
Saxony-Anhalt	7	1.4
Schleswig-Holstein	17	3.4
Thuringia	15	3.0
< 5,000 inhabitants	74	14.8
5,000–19,999 inhabitants	144	28.8
20,000–99,999 inhabitants	116	23.2
100,000–499,999 inhabitants	73	14.6
≥ 500,000 inhabitants	93	18.6

*N* = 500

### Perceptions of medical responsibility for climate change

Agreement that climate change constitutes a medical responsibility did not differ significantly across federal states, *F*(15, 500) = 0.92, *p* = .544, or practice settings, *F*(3, 500) = 0.91, *p* = .435. Similarly, no significant differences were observed across community size, *F*(4, 500) = 0.67, *p* = .616. In contrast, women (*M* = 3.94) reported significantly higher agreement than men (*M* = 3.43), *t*(445.64) = 4.31, *p* < .001, *r* = .20, *n* = 496, indicating a small to moderate effect according to Cohen [41]. Age, years of professional experience, and community size were not significantly associated with agreement (all *p’*s > 0.05).

### Physician–patient communication on climate and health

Across the sample, 62 physicians (12.4%) reported that they never addressed the topic of climate change and health in their practice, whereas the majority indicated at least occasional engagement, most commonly on a weekly (22.8%) or monthly (18.6%) basis. At the other end of the spectrum, 67 physicians (13.4%) reported discussing the topic multiple times per day (see Table 2). 63 GPs (12.6%) perceived patients as never bringing up climate-related health issues, while a considerable proportion indicated that patients raised the topic weekly (21.2%) or monthly (19.2%). Notably, 42 GPs (8.4%) indicated that patients mentioned the topic multiple times per day. Overall, both physician- and patient-initiated discussions showed a broad distribution of frequencies, with most reports clustering around weekly to monthly intervals (see Table 2).

**Table 2:** Frequencies of Discussions on Climate Change and Health

Frequency	Patient-initiated		Physician-initiated	
	*n*	%	*n*	%
Several times a day	42	8.4	67	13.4
Once a day	52	10.4	68	13.6
Once a week	106	21.2	114	22.8
Once a month	96	19.2	93	18.6
Once per quarter	42	8.4	42	8.4
Once every six months	35	7.0	17	3.4
Once a year	21	4.2	14	2.8
Less than once a year	43	8.6	23	4.6
Never	63	12.6	62	12.4

*N* = 500

### Attitudes toward ecological considerations in medical practice

**Fig. 1:**
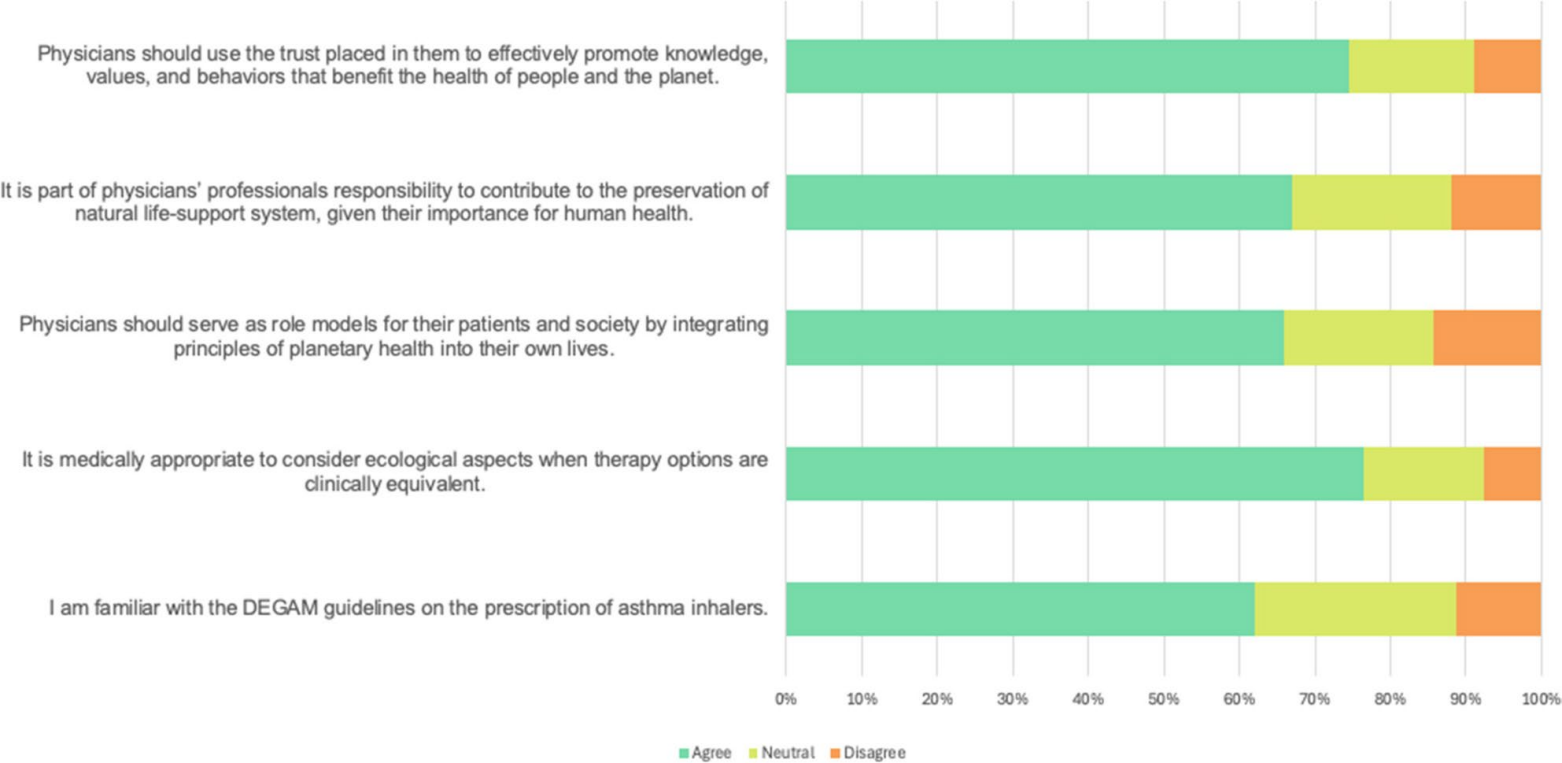
Physicians’ Responses to Items on Ecological Considerations, Professional Responsibility, and Ethical Expectations in General Practice

In response to the asthma inhaler vignette, 306 physicians (62.0%) reported familiarity with the corresponding German clinical practice guideline [42], 56 (11.3%) disagreed, and 132 (26.7%) remained neutral (*n* = 494; Fig. 1. Physicians’ responses to items on ecological considerations, professional responsibility, and ethical expectations in general practice). When asked whether it is medically appropriate to consider ecological aspects in the case that therapy options are clinically equivalent, 381 of 498 physicians (76.5%) agreed, whereas 38 (7.6%) disagreed and 79 (15.9%) expressed a neutral stance (Fig. 1).

### Attributions of Responsibility for Sustainability in Health Care

**Fig. 2:**
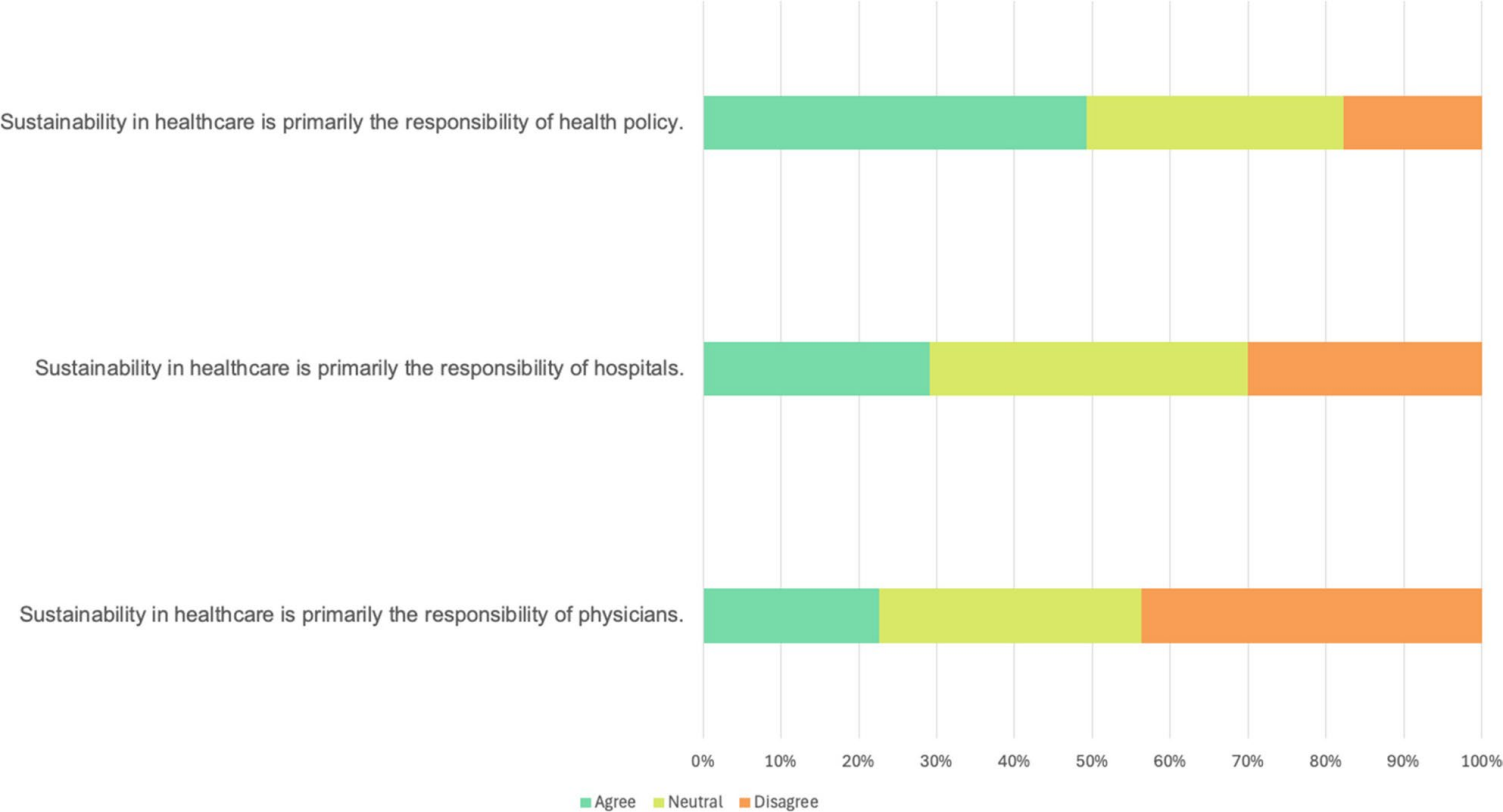
Physicians’ Attribution of Responsibility for Sustainability in Healthcare

When asked to whom responsibility for sustainability in health care should primarily be attributed, respondents differentiated clearly between institutional and individual actors. Nearly half of the physicians agreed that sustainability in health care is primarily the responsibility of health policy, with 243 of 494 participants (49.2%) endorsing this view, while 88 (17.8%) disagreed and 163 (33.0%) expressed a neutral position (Fig. 2). Regarding the role of hospitals, 144 physicians (29.1%) agreed that sustainability is mainly a task of health care institutions, whereas 148 (30.0%) disagreed and 202 (40.9%) selected a neutral response. In contrast, attributing primary responsibility to physicians themselves received the least support. Only 112 of 496 respondents (22.6%) agreed with this statement, while a substantial proportion disagreed (*n* = 217; 43.8%) or remained neutral (*n* = 167; 33.7%; Fig. 2).

### Professional responsibility and ethical expectations regarding planetary health

Across several items assessing physicians’ ethical attitudes toward ecological responsibility in medical practice, the majority expressed agreement with statements reflecting a broader professional mandate in planetary health (Fig. 1). Most respondents endorsed the idea that physicians should use the trust placed in them to promote knowledge, values, and behaviors that benefit both human and planetary health: 371 of 500 participants (74.5%) agreed, whereas 44 (8.8%) disagreed and 83 (16.7%) reported neutrality. A similar pattern emerged regarding the responsibility to help safeguard natural life-support systems due to their relevance for human health. Here, 334 of 499 physicians (67.0%) indicated agreement, 60 (12.0%) disagreed, and 105 (21.0%) expressed a neutral stance. Perceptions of physicians as societal role models were likewise prevalent: 327 of 497 participants (65.8%) agreed that physicians should exemplify principles of planetary health in their own lives, while 71 (14.3%) disagreed and 99 (19.9%) selected a neutral response.

### Associations between key items

Familiarity with the DEGAM and DGP guideline (as assessed in the asthma inhaler vignette) was positively associated with reporting that ecological consequences are discussed in practice, *r* = .25, *p* < .001, *n* = 492, representing a moderate effect [41]. Furthermore, agreement that sustainability is a medical responsibility was strongly correlated with agreement that physicians should act as role models and integrate sustainability into their own lives, *r* = .44, *p* < .001, *n* = 493, reflecting a large effect [41].

## Discussion

The present study indicates that the attribution of medical responsibility in the context of climate change exhibits a highly consistent pattern across different subgroups of GPs. The findings show that neither federal state nor community size, practice type, nor professional experience was associated with meaningful variation in agreement. This contrasts with evidence from population-based studies, including a recent large-scale German survey, which reported modest age-related demographic differences in perceptions of climate-related risks [43], as well as qualitative research indicating stronger environmental engagement among younger age groups [44]. The absence of age-related demographic differences in the present study suggests that climate-related responsibility is more uniformly anchored within the medical profession than within the general population. This degree of professional homogeneity may not be fully captured by population-based surveys and highlights the added value of profession-specific analyses. The gender disparity observed in this study aligns with established findings, in which female respondents consistently report higher ecological concern and risk sensitivity [43]. By demonstrating this pattern within a professional sample, the present study contributes to the emerging body of evidence on how demographic factors shape climate-related attitudes in healthcare settings. While population studies primarily capture societal perceptions, the present results indicate that GPs also consider climate change to be part of their professional responsibility. Overall, climate change was widely recognized as a relevant health issue and was associated with broad endorsement of professional responsibility to engage. This perspective is consistent with contemporary planetary health frameworks that emphasize the integration of climate-related responsibilities into professional standards and ethical principles [24]. These findings can be interpreted in light of established ethical frameworks, such as the Planetary Health Pledge, which highlight both individual and structural dimensions of responsibility in addressing climate change in healthcare.

Compared with international research among healthcare professionals, the present findings are highly consistent with patterns reported in other healthcare professional contexts. International survey data indicate that physicians and other healthcare professionals widely acknowledge climate change as a relevant health issue and express a general willingness to engage, while at the same time attributing primary responsibility for climate mitigation and adaptation to institutional and policy-level actors rather than to individual clinicians [31]. Our findings indicate that responsibility for sustainability is predominantly attributed to institutional and policy-level actors rather than to individual physicians. Similarly, a recent French multicenter study among healthcare professionals reported strong motivation to address environmental sustainability in healthcare alongside perceived structural barriers such as limited time, resources, and institutional support [32], which offers a plausible contextual explanation for the comparatively moderate endorsement of individual responsibility observed in the present sample. A comprehensive international review further suggests that discrepancies between normative endorsement and practical engagement are often driven by structural, educational, and institutional constraints rather than by a lack of concern among healthcare professionals [45]. In addition, an ethics-focused national survey among U.S. physicians indicates that climate-related responsibility may be acknowledged in principle, whereas acceptance of clinical trade-offs remains cautious and appears to depend on institutional frameworks and guidance [36], paralleling the ambivalence observed in the vignette-based component of the present study. Overall, these studies support the interpretation that within professional samples, attitudes are shaped more by shared norms and structural conditions than by many demographic or practice characteristics, with some subgroup differences (e.g., gender) still occurring.

Our results further indicate that environmental and climate-related issues are a recurrent topic in the clinical routine of general practice. The fact that these issues are raised on a regular basis by both general practitioners and patients suggests that both sides consider the topic to be appropriate for the healthcare setting. Climate-related health concerns are therefore recognized as a relevant aspect of medical consultations. In addition, the regular discussion of environmental issues initiated by both parties indicates that climate-related questions extend beyond an abstract level. Instead, particularly in the context of minor or low-risk conditions, there is growing acceptance in the general population that climate-sensitive considerations may legitimately inform therapeutic decision-making [37]. The observation that patients proactively engage in environmental issues can be interpreted as an indication that medical professionals are perceived as legitimate sources of information and support in this domain. This suggests that primary care may represent a relevant setting for health-related climate communication and points to the relevance of integrating such content into continuing education, clinical guidelines, and patient counseling [6].

The present results indicate that the majority of GPs surveyed are open to considering ecological aspects in their clinical practice when different treatment options do not differ in terms of expected clinical outcomes. At the same time, a considerable proportion of participants adopts a neutral stance or does not unequivocally endorse ecological considerations having an impact on treatment decision making. This pattern may point to a tension between general agreement with the need for environmental protection and uncertainty regarding its normative status in clinical practice. This ambivalence is also evident in the clinical vignette, which addressed the consideration of ecological aspects in situations involving clinically equivalent treatment options. The present data do not allow for conclusions about the underlying reasons for this ambivalence. Professional codes of conduct currently address aspects related to environmental sustainability only to a limited extent. Against this background, the observed ambivalence can be situated within a professional context in which ecological responsibility has not yet been consistently articulated within standards such as clinical practice guidelines, leaving limited normative orientation for everyday clinical decision-making. This pattern suggests that while a majority of respondents endorse ecological considerations in situations of clinical equivalence, a substantial proportion remains uncertain or neutral.

Ethical conflicts may arise if individual clinical outcomes need to be weighed against environmental protection. Although professional ethical codes encompass general obligations to avoid harm, use resources responsibly, and prioritize the well-being of patients and society, they often provide limited operational guidance on how to address ecological conflicts of interest in clinical practice [28]. The ambivalence observed in this study may therefore be understood less as a rejection of ecological responsibility and more as an expression of a lack of normative clarity. Consequently, the findings do not necessitate a radical transformation of physicians’ professional obligations. Rather, they suggest that existing professional codes could be specified more clearly to provide explicit ethical guidance in situations where alternative treatment options are medically equivalent in terms of expected clinical outcomes. The provision of such clarification has the potential to mitigate uncertainty without imposing undue restrictions on professional discretion.

Beyond professional codes of ethics, the present results indicate that GPs associate ecological and climate-related issues with their professional self-understanding. This orientation is reflected in respondents’ strong support (65.8%) for the idea that medical professionals should serve as role models by promoting principles of planetary health in both their professional and personal contexts. At the same time, the findings suggest that GPs do not perceive themselves as the primary agents responsible for implementing climate protection measures in healthcare. Instead, responsibility is more frequently attributed to the meso and macro levels, such as healthcare institutions, professional bodies, or health policy frameworks. Against this background, the emphasis on role modeling can be interpreted as an expression of openness and acceptance at the level of everyday clinical practice (micro level), while acknowledging that meaningful implementation is perceived to require action and support at meso- and macro-levels.

A major strength of the present study is the large sample size combined with a nationwide survey, which allowed for the inclusion of a structurally heterogeneous group of general practitioners across different practice settings. The focus on a clearly defined target population—GPs—enhances the relevance and interpretability of the findings for primary care. Furthermore, the study simultaneously addressed attitudinal, normative, and practice-related dimensions of climate protection, capturing not only physicians’ evaluations of climate action but also its perceived normative relevance and its presence in everyday clinical practice. The use of a realistic, general-practice–typical case vignette further increased ecological validity. In addition, the questionnaire was developed in a multiprofessional process involving GPs, ethicists, and psychologists, ensuring both clinical relevance and conceptual breadth.

Some limitations should be acknowledged. The exploratory nature of the study design should be considered when interpreting the findings. The cross-sectional design precludes causal inferences regarding the observed associations. Recruitment via the DocCheck panel may have introduced a selection bias, potentially leading to an overrepresentation of physicians who are more digitally affine or particularly interested in climate-related topics. The recoding of five-point Likert scales into three categories may have resulted in a loss of variance and reduced sensitivity. In addition, the exclusive reliance on self-reported data without behavioral or observational measures introduces the risk of social desirability bias and limits the objectivity of the findings. Although the questionnaire was developed in a multiprofessional process and reviewed by general practitioners, it did not undergo formal psychometric validation; therefore, reliability and validity in the strict test-psychological sense cannot be fully assured. Moreover, the gender distribution of the sample deviates from national physician statistics, with male GPs being overrepresented (38.2% in the present study vs. approximately 65% female among GPs nationwide), whereas the mean age of participants closely matches national averages [39]. This imbalance may limit the generalizability of the findings, particularly with regard to gender-related differences in attitudes toward climate and sustainability issues. Finally, individual interpretations of key concepts such as *sustainability* or *climate change* may vary among participants, which could further constrain the construct validity of the assessed variables.

Against this background, the implications of our findings extend to stakeholders within the healthcare system. For GPs, the findings indicate broad support for a role-model function in relation to climate and environmental issues. This endorsement provides an empirical basis for integrating climate-sensitive considerations into appropriate clinical decision-making contexts. At the level of professional organizations, including medical associations and guideline commissions, the findings suggest that climate-related initiatives are likely to encounter a receptive professional audience. This facilitates the integration of climate-sensitive considerations into professional standards, particularly in clinical practice guidelines. At the health policy level, the results suggest that responsibility attribution extends beyond individual physicians to professional, organizational, and policy-related actors, highlighting the role of structural incentives in supporting climate-sensitive counselling. In relation to patients, the findings suggest that climate-sensitive concerns are likely to be met with openness among a substantial proportion of GPs, underscoring that these topics can be addressed within routine primary care consultations without encountering substantial resistance.

Implications also extend to medical training. In medical education, early engagement with planetary health and responsibility-related questions may support future physicians in navigating normative tensions without framing ecological responsibility as an additional individual obligation only. In advanced trainings the focus should be laid on providing physicians with an evidential basis on the ecological impacts of interventions within their field of expertise and to further sensitize them towards appropriate ways of communicating such issues in clinical practice. This could help to avoid physicians eschewing ecological topics in clinical consultation out of ignorance of the facts or uncertainty how to appropriately communicate them.

Further research is needed in several areas. First, more systematic empirical evidence is needed to assess the environmental impact of concrete medical practices and treatment options and to clarify how such evidence may be explicitly incorporated into clinical practice guidelines. Second, building on this evidence base, future studies should examine the knowledge level and educational needs of GPs with regard to climate- and environment-related aspects of medical care, including how professional guidance is interpreted and applied in everyday practice. Finally, qualitative research focusing on the content of clinical consultations could complement these findings by providing more detailed insights into which climate-related issues are considered relevant by physicians and patients and how different considerations are perceived and accepted in routine care. In addition, future research could further draw on ethical frameworks, such as the Planetary Health Pledge, to better understand how normative concepts of responsibility translate into clinical practice.

## Conclusions

The present study provides one of the first nationwide quantitative insights into how GPs in Germany situate climate- and environment-related issues within their professional self-understanding, normative orientations, and clinical practice. The findings indicate a differentiated pattern in which broad normative endorsement of climate-related concerns coexists with more cautious attributions of individual responsibility. Overall, the results suggest that climate- and environment-related issues are recognized as relevant in general practice, while responsibility is perceived as extending beyond individual physicians to broader institutional and policy-level contexts.

## Supplementary Information

Supplementary Material 1.

## Data Availability

The datasets generated and analyzed during the current study are available from the corresponding author on reasonable request.

## References

[1] Haines A, Ebi K. The imperative for climate action to protect health. N Engl J Med. 2019;380:263–73. 30650330 10.1056/NEJMra1807873

[2] Ebi KL, Ogden NH, Semenza JC, Woodward A. Detecting and attributing health burdens to climate change. Environ Health Perspect. 2017;125:085004. 28796635 10.1289/EHP1509 PMC5783629

[3] Mitchell D, Heaviside C, Vardoulakis S, Huntingford C, Masato G, et al. Attributing human mortality during extreme heat waves to anthropogenic climate change. Environ Res Lett. 2016;11:074006.

[4] Romanello M, Walawender M, Hsu S et al. The 2025 report of the Lancet Countdown on health and climate change: climate change action offers a lifeline. The Lancet. 2025;406:2804–57. 10.1016/S0140-6736(25)01919-1 41175887

[5] Burrows K, Denckla CA, Hahn J, Schiff JE, Okuzono SS, Randriamady H, et al. A systematic review of the effects of chronic, slow-onset climate change on mental health. Nat Ment Health. 2024;2:228–43. 41098556 10.1038/s44220-023-00170-5 PMC12520228

[6] World Health Organization. COP26 special report on climate change and health: the health argument for climate action [Internet]. 2021. Available from: https://iris.who.int/server/api/core/bitstreams/ec426159-c2b9-436f-95bf-505918522e0d/content. Accessed 4 Feb 2026.

[7] Or Z, Seppänen A-V. The role of the health sector in tackling climate change: a narrative review. Health Policy. 2024;143:105053. 38537397 10.1016/j.healthpol.2024.105053

[8] Greenhouse Gas Protocol. A Corporate Accounting and Reporting Standard [Internet]. Available from: https://ghgprotocol.org/sites/default/files/standards/ghg-protocol-revised.pdf. Accessed 4 Feb 2026.

[9] Lattanzio S, Stefanizzi P, D’Ambrosio M, Cuscianna E, Riformato G, Migliore G, et al. Waste management and the perspective of a green hospital-a systematic narrative review. Int J Environ Res Public Health. 2022;19:15812. 36497884 10.3390/ijerph192315812 PMC9738387

[10] Houziel C, Prothon E, Trinh-Duc A. Carbon footprint of general practice: Retrospective case study of GP offices in a rural department of France. Clim Change Health. 2023;14:100273.

[11] Nicolet J, Mueller Y, Paruta P, Boucher J, Senn N. What is the carbon footprint of primary care practices? A retrospective life-cycle analysis in Switzerland. Environ Health. 2022;21:3. 34980135 10.1186/s12940-021-00814-y PMC8723904

[12] NAPRA. Treibhausgasemissionen im Gesundheitssektor [Internet]. 2025. Available from: https://www.napra.info/hintergrund. Accessed 4 Feb 2026.

[13] NHS England. Delivering a net zero NHS [Internet]. Available from: https://www.england.nhs.uk/greenernhs/a-net-zero-nhs/. Accessed 4 Feb 2026.

[14] Schmidt L, Bohnet-Joschko S. Planetary health and hospitals’ contribution-a scoping review. Int J Environ Res Public Health. 2022;19:13536. 36294116 10.3390/ijerph192013536 PMC9603437

[15] Schwab R, Schiestl LJ, Hasenburg A. Greening the future of healthcare: implementation of sustainability strategies in German hospitals and beyond—a review. Front Public Health. 2025;13:1559132. 40385621 10.3389/fpubh.2025.1559132 PMC12082835

[16] Moyo TN, C P, Mashingaidze TM. Multi-level governance and climate change adaptation: A systematic literature review. Sustainable Futures. 2026;11:101724.

[17] Sandler R. Ethical Theory and the Problem of Inconsequentialism: Why Environmental Ethicists Should be Virtue-Oriented Ethicists. J Agric Environ Ethics. 2010;23:167–83.

[18] Sinnott-Armstrong W. It’s Not *My* Fault: Global Warming and Individual Moral Obligations. Climate Ethics: Essential Readings (New York, 2010; online edn, Oxford Academic, 12 Nov. 2020). 10.1093/oso/9780195399622.003.0029. Accessed 6 June 2026.

[19] Richie C, Samuel G. Who carries the responsibility for health care carbon reduction? Hastings Cent Rep. 2025;55:7–14. 10.1002/hast.5008 PMC12207287 40557913

[20] Heidbrink L. Nichtverantwortlichkeit: Zur Deresponsibilisierung der Gesellschaft. Weilerswist: Velbrück Wissenschaft; 2024.

[21] Wild V. A bio-psycho-socio-planetary model of health and Iris Marion Young’s concept of responsibility as foundation for a medical ethos during environmental crises. GMS J Med Educ. 2026;43(3):Doc37. 41953158 10.3205/zma001831 PMC13054810

[22] Richie C. Climate-relation actions for health care systems-not professionals-are obligatory. Am J Bioeth. 2025;25:46–50. 10.1080/15265161.2025.2509947 40622791

[23] Resnik DB, Pugh J. Green bioethics, patient autonomy and informed consent in healthcare. J Med Ethics. 2024;50:489–93. 37833040 10.1136/jme-2023-109404 PMC11014890

[24] Wabnitz K-J, Gabrysch S, Guinto R, Haines A, Herrmann M, Howard C, et al. A pledge for planetary health to unite health professionals in the Anthropocene. Lancet. 2020;396:1471–3. 33010210 10.1016/S0140-6736(20)32039-0 PMC7527204

[25] Wiesing U. Climate change and the different roles of physicians: a critical response to A planetary health pledge for health professionals in the Anthropocene. Med Health Care Philos. 2022;25:161–4. 34529217 10.1007/s11019-021-10051-2 PMC8443911

[26] van Gils-Schmidt HJ, Salloch S. Physicians’ duty to climate protection as an expression of their professional identity: a defence from Korsgaard’s neo-Kantian moral framework. J Med Ethics. 2024;50:368–74. 37879902 10.1136/jme-2023-109203

[27] Hantel A, Marron JM, Abel GA. Establishing and defining an approach to climate conscious clinical medical ethics. Am J Bioeth. 2025;25:8–21. 38635462 10.1080/15265161.2024.2337418 PMC11486837

[28] Richie C. Green informed consent in the classroom, clinic, and consultation room. J Med Ethics. 2023;26:507–15. 10.1007/s11019-023-10163-x PMC10725850 37584839

[29] Salloch S. Ecological preferences and patient autonomy. J Med Ethics. 2025;51(9):609–13. 39638547 10.1136/jme-2024-110432 PMC12418574

[30] Kuiter SG, Herrmann A, Mertz M, Quitmann C, Salloch S. Should healthcare professionals include aspects of environmental sustainability in clinical decision-making? A systematic review of reasons. BMC Med Ethics. 2025;26:78. 40611029 10.1186/s12910-025-01230-4 PMC12226885

[31] Kotcher J, Maibach E, Miller J, Campbell E, Alqodmani L, Maiero M, et al. Views of health professionals on climate change and health: a multinational survey study. Lancet Planet Health. 2021;5:e316–23. 33838130 10.1016/S2542-5196(21)00053-X PMC8099728

[32] Guihenneuc J, Cambien G, Blanc-Petitjean P, Papin E, Bernard N, Jourdain B, et al. Knowledge, behaviours, practices, and expectations regarding climate change and environmental sustainability among health workers in France: a multicentre, cross-sectional study. Lancet Planet Health. 2024;8:e353–64. 38849178 10.1016/S2542-5196(24)00099-8

[33] André H, Gonzalez Holguera J, Depoux A, Pasquier J, Haller DM, Rodondi P-Y, et al. Talking about Climate Change and Environmental Degradation with Patients in Primary Care: A Cross-Sectional Survey on Knowledge, Potential Domains of Action and Points of View of General Practitioners. Int J Environ Res Public Health. 2022;19:4901. 35457768 10.3390/ijerph19084901 PMC9029888

[34] Albrecht L, Reismann L, Leitzmann M, Bernardi C, von Sommoggy J, Weber A, Jochem C. Climate-specific health literacy in health professionals: an exploratory study. Front Med (Lausanne). 2023;10:1236319. 37928468 10.3389/fmed.2023.1236319 PMC10622978

[35] Quitmann C, Sauerborn R, Danquah I, Herrmann A. Climate change mitigation is a hot topic, but not when it comes to hospitals: a qualitative study on hospital stakeholders’ perception and sense of responsibility for greenhouse gas emissions. J Med Ethics. 2023;49:204–10. 35459742 10.1136/medethics-2021-107971 PMC9985738

[36] Hantel A, Senay E, Hlubocky FJ, Walsh TP, Johnston H, Cronin A, et al. The ethics of climate change and health-care delivery: a national survey of US-based physicians. Lancet Planet Health. 2025;9:101289. 40812328 10.1016/j.lanplh.2025.101289

[37] van Bree EM, van Gestel LC, Visser EH, Aardoom JJ, Brakema EA, Adriaanse MA. Integrating environmental sustainability in clinical counselling: a randomised, double-blind, experimental vignette study in the Netherlands. Lancet Planet Health. 2025;9(11):101328. 10.1016/j.lanplh.2025.101328 41317741

[38] Herrmann A, Krippl N, Fischer H, Nieder J, Griesel S, Bärnighausen T, et al. Acceptability of health-only versus climate-and-health framings in lifestyle-related climate-sensitive health counselling: results of a randomised survey experiment in Germany. Lancet Planet Health. 2025;9:e456–66. 40516537 10.1016/S2542-5196(25)00110-X

[39] Bundesärztekammer. Ärztestatistik zum 31. Dezember 2024. 2024. Available from: https://www.bundesaerztekammer.de/fileadmin/user_upload/BAEK/Ueber_uns/Statistik/AErztestatistik_2024.pdf. Accessed 4 Feb 2026.

[40] Faul F, Erdfelder E, Buchner A, Lang A-G. Statistical power analyses using G*Power 3.1: tests for correlation and regression analyses. Behav Res Methods. 2009;41:1149–60. 19897823 10.3758/BRM.41.4.1149

[41] Cohen J. A power primer. Psychol Bull. 1992;112:155–9. 19565683 10.1037//0033-2909.112.1.155

[42] DEGAM DGP. S2k-Leitlinie Klimabewußte Verordnung von Inhalativa [Internet]. 2024. Available from: https://register.awmf.org/de/leitlinien/detail/053-059. Accessed 4 Feb 2026.

[43] German Environment Agency. Environmental Awareness in Germany 2024 [Internet]. 2025. Available from: https://www.umweltbundesamt.de/sites/default/files/medien/479/publikationen/uba_ubs24_shortreport.pdf. Accessed 4 Feb 2026.

[44] Boermans DD, Jagoda A, Lemiski D, Wegener J, Krzywonos M. Environmental awareness and sustainable behavior of respondents in Germany, the Netherlands and Poland: A qualitative focus group study. J Environ Manage. 2024;370:122515. 39299123 10.1016/j.jenvman.2024.122515

[45] Uppalapati S, Campbell J, Kotcher EW, Maibach E et al. Public and health professional engagement with climate change as a health issue: a review of the literature [Internet]. SSRN; 2023. Available from: https://ssrn.com/abstract=4536308. Accessed 4 Feb 2026.

