# Supplementary material for: Climate change and professional responsibility: a cross-sectional survey among German general practitioners
Source: BMC Med Ethics. 2026 Jun 9;27:105. doi: 10.1186/s12910-026-01516-1 (PMC13248457; doi:10.1186/s12910-026-01516-1)

**Socio-demographic data:**

- How old are you?
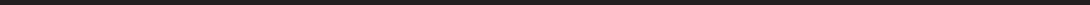
years
- Gender:

female

male

diverse

no answer

- Federal state in which you practice medicine:

Baden-Württemberg

Bavaria

Berlin

Brandenburg

Bremen

Hamburg

Hesse

Mecklenburg-Vorpommern

Lower Saxony

North Rhine-Westphalia

Rhineland-Palatinate

Saarland

Saxony

Saxony-Anhalt

Schleswig-Holstein

Thuringia

- Size of the municipality in which you practice medicine:

under 5,000 inhabitants

5,000-20,000 inhabitants

20,000-100,000 inhabitants

100,000-500,000 inhabitants

over 500,000 inhabitants

- What type of practice do you work in?

solo practice

joint practice

practice collective

medical care center

other

How many years have you been practicing medicine?
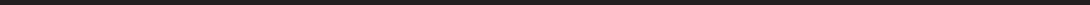
years

**Likert scale:**

| Strongly disagree |  |  |  | Strongly agree | No opinion |
| --- | --- | --- | --- | --- | --- |
| 1 | 2 | 3 | 4 | 5 |  |

**In the following, a clinical case from routine practice is presented. You are then asked to evaluate the statements below:**

Mr. T., 68 years old, has been living for several years with well-controlled bronchial asthma. You have been providing care for him in your practice for 8 years. He is very familiar with the use of his metered-dose inhaler (MDI). At your most recent continuing medical education course, you learned that MDIs release substantially higher amounts of climate-damaging greenhouse gases compared with dry powder inhalers (DPIs).

From a medical perspective, a DPI would also be a suitable option for Mr. T.; however, he would need to relearn the inhalation technique. It is unclear how receptive he would be to such a change, as he has often been skeptical toward changes in the past.

**How do you evaluate the following statements?**

*The following items were assessed using a 5-Point-Likert-Scale*

I would actively address the environmental impact of different inhalers in the conversation with the patient.

I would feel supported in such decisions by recommendations from medical professional societies.

**Ecological Considerations in Clinical Decision-Making**

I consider it part of my professional responsibility as a physician to inform patients about more environmentally friendly treatment options, even without a specific request.

It is medically appropriate to consider ecological aspects when therapy options are clinically equivalent.

I am familiar with the DEGAM guidelines on the prescription of asthma inhalers.

I do not consider climate protection to be part of my professional role as a physician.

**Frequency of Climate- and Health-Related Discussions in Clinical Practice**

How often are topics related to climate change and health raised by patients themselves in your practice, approximately?

several times a day

once a day

once a week

once a month

once per quarter

once every six months

once a year

less than once a year

never

Which topics related to climate change and health do patients raise?


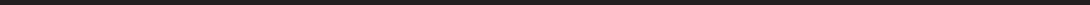


How often do you yourself (as a physician) address topics related to climate change and health in your practice, approximately?

several times a day

once a day

once a week

once a month

once per quarter

once every six months

once a year

less than once a year

never

Which topics do you address?


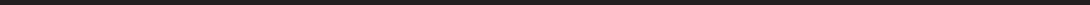


**Attribution of responsibility for sustainability in healthcare.**

*The following items were assessed using a 5-Point-Likert-Scale*

Sustainability in healthcare is primarily the responsibility of health policy.

Sustainability in healthcare is primarily the responsibility of hospitals.

Sustainability in healthcare is primarily the responsibility of physicians.

**Professional Advocacy for Climate Protection in Healthcare**

Physicians should publicly advocate for stronger climate protection in healthcare.

Physicians should advocate for stronger climate protection in healthcare within their professional organizations (e.g., medical chambers or professional associations).

**Normative Professional Responsibilities Related to Planetary Health**

It is part of physicians’ professionals responsibility to contribute to the preservation of natural life-support system, given their importance for human health.

Physicians should use the trust placed in them to effectively promote knowledge, values, and behaviors that benefit the health of people and the planet.

Physicians should serve as role models for their patients and society by integrating principles of planetary health into their own lives.

**Education and Professional Boundaries Regarding Climate Responsibility**

Climate protection should be addressed more strongly in undergraduate, postgraduate, and continuing medical education.

I reject integrating climate and environmental responsibility into my role as a physician.

Which additional aspects related to climate protection and healthcare do you consider important?


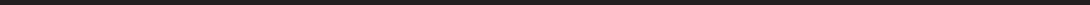

Supplement: Supplementary file 1 — Supplementary Material 1. [file 12910_2026_1516_MOESM1_ESM.docx]
